# Synaptic Vesicle Disruption in Parkinson’s Disease: Dual Roles of α-Synuclein and Emerging Therapeutic Targets

**DOI:** 10.3390/brainsci16010007

**Published:** 2025-12-20

**Authors:** Mario Treviño, Magdalena Guerra-Crespo, Francisco J. Padilla-Godínez, Emmanuel Ortega-Robles, Oscar Arias-Carrión

**Affiliations:** 1 Laboratorio de Plasticidad Cortical y Aprendizaje Perceptual, Instituto de Neurociencias, Universidad de Guadalajara, Guadalajara 44130, Mexico; 2 Laboratory of Regenerative Medicine, Department of Physiology, Faculty of Medicine, National Autonomous University of Mexico, Mexico City 04510, Mexico; mguerra@facmed.unam.mx; 3 Department of Mathematics and Physics, Western Institute of Technology and Higher Education, Tlaquepaque 45604, Mexico; franciscoj.padilla@iteso.mx; 4 División de Neurociencias, Clínica, Instituto Nacional de Rehabilitación Luis Guillermo Ibarra Ibarra, Mexico City 14389, Mexico; edortegar@gmail.com; 5 Tecnologico de Monterrey, Escuela de Medicina y Ciencias de la Salud, Mexico City 14380, Mexico

**Keywords:** Parkinson’s disease, α-synuclein, synaptic vesicles, SNARE complex, lipidomics, vATPase, presynaptic dysfunction, neurodegeneration, drug discovery

## Abstract

Evidence increasingly indicates that synaptic vesicle dysfunction emerges early in Parkinson’s disease (PD), preceding overt dopaminergic neuron loss rather than arising solely as a downstream consequence of neurodegeneration. α-Synuclein (αSyn), a presynaptic protein that regulates vesicle clustering, trafficking, and neurotransmitter release under physiological conditions, exhibits dose-, conformation-, and context-dependent actions that distinguish its normal regulatory roles from pathological effects observed in disease models. This narrative review synthesizes findings from a structured search of PubMed and Scopus, with emphasis on α-syn-knockout (αSynKO) and BAC transgenic (αSynBAC) mouse models, which do not recapitulate the full human PD trajectory but provide complementary insights into αSyn physiological function and dosage-sensitive vulnerability. Priority was given to studies integrating ultrastructural approaches—such as cryo-electron tomography, high-pressure freezing/freeze-substitution TEM, and super-resolution microscopy—with proteomic and lipidomic analyses. Across these methodologies, several convergent presynaptic alterations are consistently observed. In vivo and ex vivo studies associate αSyn perturbation with impaired vesicle acidification, consistent with altered expression or composition of vacuolar-type H^+^-ATPase subunits. Lipidomic analyses reveal age- and genotype-dependent remodeling of vesicle membrane lipids, particularly curvature- and charge-sensitive phospholipids, which may destabilize αSyn–membrane interactions. Complementary biochemical and cell-based studies support disruption of SNARE complex assembly and nanoscale release-site organization, while ultrastructural analyses demonstrate reduced vesicle docking, altered active zone geometry, and vesicle pool disorganization, collectively indicating compromised presynaptic efficiency. These findings support a synapse-centered framework in which presynaptic dysfunction represents an early and mechanistically relevant feature of PD. Rather than advocating αSyn elimination, emerging therapeutic concepts emphasize preservation of physiological vesicle function—through modulation of vesicle acidification, SNARE interactions, or membrane lipid homeostasis. Although such strategies remain exploratory, they identify the presynaptic terminal as a potential window for early intervention aimed at maintaining synaptic resilience and delaying functional decline in PD.

## 1. Introduction

Synaptic vesicles (SVs) are the core units of neurotransmission, enabling storage, activity-dependent release, and recycling of neurotransmitters at presynaptic terminals. Although ultrastructurally uniform, SVs exhibit marked molecular heterogeneity, influenced by neuronal subtype, activity state, and developmental stage, thereby shaping rapid, precise synaptic signaling [1,2]. However, the functional integrity of this system becomes increasingly vulnerable with age, as vesicle dynamics slow, neurotransmitter gradients falter, and compensatory mechanisms erode [3,4]. In Parkinson’s disease (PD) and other neurodegenerative disorders, synaptic vesicle vulnerabilities are markedly amplified, yet the precise mechanisms linking SV dysfunction to disease initiation and progression remain poorly understood [5,6].

Emerging evidence suggests that presynaptic failure is not merely a downstream consequence of neuronal degeneration in PD, but rather an early and potentially initiating event [7]. Multiple lines of data (including transcriptomic, proteomic, and ultrastructural studies) indicate that synaptic abnormalities precede dopaminergic cell loss [8]. Genetic associations reinforce this view: key PD risk genes such as SNCA, LRRK2, DNAJC6, SYNJ1, and SH3GL2 encode proteins involved in vesicle trafficking, lipid regulation, and membrane curvature [9]. These findings implicate synaptic vesicle biology not only in disease pathogenesis but also in the selective vulnerability of midbrain neurons [5]. Nevertheless, much of this evidence derives from associative molecular signatures or ex vivo tissue, and clear causal pathways linking early synaptic changes to subsequent neurodegeneration remain incompletely defined. Interpretation is further complicated by methodological constraints, including reliance on αSyn overexpression models that do not reproduce Lewy pathology or progressive nigrostriatal degeneration, and human tissue sampled at end-stage disease, which obscures early mechanistic steps [10,11,12].

This review aims to characterize α-synuclein (αSyn) not merely as a pathological substrate, but as a key structural and functional regulator of synaptic vesicle integrity [10,12]. Under physiological conditions, α-synuclein binds to negatively charged phospholipids on the vesicle membrane, promotes clustering, and facilitates soluble N-ethylmaleimide-sensitive factor attachment protein receptor (SNARE) complex assembly through interactions with VAMP2 [6,13]. These functions depend on a delicate balance of concentration, membrane affinity, and conformational state. Disruption of this balance, whether through misfolding, an altered lipid environment, or excess cytosolic accumulation, can destabilize the SNARE machinery, alter vesicle pool organization, and produce defects in vesicle acidification and recycling, ultimately compromising neurotransmitter release [8]. Importantly, these defects can occur independently of protein aggregation, highlighting a model in which both loss of physiological function and toxic gain-of-function converge to destabilize the presynaptic terminal [14]. However, many of these mechanistic links derive from in vitro or ex vivo experiments; direct in vivo causal evidence remains more limited.

Given the early convergence of α-synuclein dysregulation and synaptic vesicle dysfunction in PD, there is growing interest in exploring whether stabilizing presynaptic mechanisms might modify disease trajectory, although definitive evidence for disease-modifying efficacy in vivo is currently lacking. Therapeutic concepts such as enhancing vATPase-mediated acidification, supporting SNARE complex stability, or correcting lipid imbalances (including phosphatidylserine and phosphatidylcholine deficits) remain exploratory and are supported largely by preclinical mechanistic studies rather than demonstrated clinical benefit [7,11,15,16]. Within this framework, the focus is on protecting physiological vesicle function rather than implying reversal of established disease or restoration of normal presynaptic architecture [11]. By integrating molecular profiling with functional validation across models of aging and α-synuclein perturbation, drug discovery in PD can move beyond symptom control and toward preservation of ‘synaptic resilience’.

## 2. Methods

To inform this expert review, we conducted a structured but non-systematic literature search of the PubMed and Scopus databases covering the period from January 2000 to February 2025. Search terms included combinations of “Parkinson’s disease”, “α-synuclein”, “synaptic vesicle”, “presynaptic dysfunction”, “SNARE complex”, “vATPase”, “lipidomics”, “neurodegeneration”, “mouse models”, and “drug discovery”. As appropriate for an expert narrative review, study selection and database filtration were performed at the authors’ discretion, guided by relevance, mechanistic depth, and consistency with the conceptual focus of the manuscript. We prioritized original research articles and high-quality reviews that provided mechanistic insights into synaptic vesicle regulation, α-synuclein physiology or pathology, and early presynaptic dysfunction, with particular emphasis on studies employing genetic mouse models (α-synuclein knockout and BAC transgenic lines), synaptosomal or vesicle-enriched preparations, and integrated ultrastructural, proteomic, lipidomic, or functional approaches. Studies were excluded if they were purely descriptive, focused exclusively on late-stage neurodegeneration without synaptic analysis, lacked mechanistic interpretation, or addressed α-synuclein biology outside a presynaptic or vesicle-related context. Additional filtration favored studies cited repeatedly across the field or supported by convergent experimental evidence. Reference lists of key articles were also examined to identify influential or foundational studies relevant to the themes developed in this review.

## 3. Ultrastructural Reprogramming of Synaptic Vesicles by α-Synuclein

SVs form a highly ordered nanoscale compartment within presynaptic terminals, where membrane curvature, lipid composition, and cytomatrix architecture converge to support fast neurotransmission [17]. Electron tomography reveals SVs as ~40–50 nm-diameter spheres arranged in concentric pools around the active zone, separated by ~10–15 nm inter-vesicle spacing and tethered by filamentous scaffolds that couple them to the release machinery [18,19]. This geometry generates an environment enriched in anionic phospholipids—particularly phosphatidylserine and phosphatidylethanolamine—whose surface curvature and charge density create a privileged binding platform for αSyn [20,21].

Within this ultrastructural landscape, αSyn acts as a curvature-sensing, amphipathic membrane binder, with its N-terminal region transitioning from disordered states in solution to extended or kinked α-helical conformations upon lipid engagement. High-resolution NMR and cryo–electron spectroscopic studies show that residues 1–60 adopt two adjacent helices separated by a dynamic hinge, allowing αSyn to interface with a single vesicle or bridge two vesicles simultaneously [22]. This “double-anchor” mechanism provides a structural model in which αSyn stabilizes vesicle clusters by binding a single vesicle via its first helix [23]. In contrast, the second helix engages either the plasma membrane or a neighboring vesicle, thereby maintaining physiological vesicle spacing and promoting the formation of stable vesicle pools [20]. Ultrastructurally, these interactions align closely with the ~10–15 nm SV–SV distances observed by tomography, suggesting that αSyn is optimally tuned to the geometry of the presynaptic vesicle cluster [18,24]. While these conformational principles come primarily from biophysical and reconstituted systems, in vivo studies corroborate a role for endogenous synuclein in maintaining vesicle clustering and docking, as shown by acute perturbation experiments at vertebrate synapses [25].

This membrane-bound architecture is exquisitely sensitive to sequence determinants. The Met-X_3_-Met motif at the extreme N-terminus serves both as a high-affinity Cu(I) binding site and a regulator of local helix stabilization [26]. Cu(I) coordinates with Met1, Asp2, and Met5, promoting α-helical content and enhancing lipid association [27]. Mutations that perturb this region—such as the PD–linked V15A variant—reduce membrane affinity without grossly altering the disordered monomeric ensemble, thereby increasing the concentration of free αSyn available for NAC-mediated aggregation [28]. This shift enhances liposome-associated fibril formation and disrupts the balance between vesicle-bound and aggregation-competent αSyn pools [29]. In parallel, aromatic residues at position Tyr-39 modulate long-range interactions with the NAC region and contribute to the structural interface for amyloid-modulating ligands. Substitutions that diminish aromaticity compact the monomer and redistribute binding surfaces, revealing a key structural node that influences both membrane binding and early aggregation events [30]. These mechanistic insights derive from biochemical and structural studies; their presynaptic consequences remain largely inferential rather than directly demonstrated in vivo.

The presynaptic consequences of these molecular perturbations are striking. When αSyn is appropriately membrane-bound, it stabilizes vesicle pools, supports SNARE complex assembly, and reinforces the tethering architecture that defines the active zone [15,25]. Direct in vivo evidence for these functions is strongest for vesicle clustering and docking [25] and for activity-dependent vesicle recycling deficits caused by excess αSyn [31], while support for SNARE facilitation comes primarily from in vitro and cellular assays. However, lipidomic shifts typical of neurodegeneration—including altered cholesterol or phosphatidylserine levels—shift the mode of αSyn binding, such that the NAC region, rather than the amphipathic N-terminus, becomes the primary membrane interface [32]. This reconfiguration disrupts vesicle docking and compromises the release machinery [21]. Although lipid depletion or remodeling can mislocalize αSyn, current evidence does not support that lipid changes alone are sufficient to drive the transition to β-rich fibrillar states; additional cellular stressors and aggregation-prone conformations are required. Overexpression or mutant forms of αSyn further impair vesicle recycling, slowing endocytic retrieval and reducing the effective number of release-competent vesicles, as demonstrated by ultrastructural and functional studies of αSyn and A53T-induced recycling defects [33]. These impairments reflect causal effects of αSyn dosage or mutation, whereas broader lipid or proteomic changes observed in synucleinopathy models represent associative signatures rather than direct mechanisms.

As αSyn disengages from its physiological helical state and accumulates in soluble or membrane-associated oligomers, the conformational ensemble shifts toward β-sheet-rich species that seed fibril formation [34]. Patient-derived αSyn fibrils display structural heterogeneity not recapitulated by any in vitro polymorph, indicating that the presynaptic ultrastructure—its lipid environment, curvature, and protein density—imprints disease-specific conformational signatures onto αSyn assemblies [35,36]. Recent cryo-EM comparisons further show that mouse αSyn fibrils differ structurally and functionally from human Lewy body–derived fibrils [37], underscoring species-specific constraints and limiting the translational reach of murine models. These findings support the idea that presynaptic membranes influence fibril morphology but do not by themselves resolve how pathogenic strains arise during human disease progression.

Together, these findings establish a unified structural model in which αSyn operates as a nanoscale architect of synaptic vesicles. Its physiological function depends on a finely tuned membrane-bound α-helical ensemble that integrates curvature sensing, vesicle bridging, and SNARE support. This model is shaped by a combination of in vivo evidence (e.g., vesicle clustering/docking) and biochemical inference (e.g., helix–hinge bridging, Cu-dependent stabilization). Disruption of this ensemble—through mutation, metal dyshomeostasis, lipid remodeling, or emerging fibrillar species—can perturb presynaptic architecture, destabilizing vesicle pools, obstructing docking and recycling, and seeding pathogenic aggregates, but the degree to which these mechanisms initiate versus amplify early synaptic defects in PD remains unresolved. This transition from membrane-scaffold protein to fibril-forming catalyst represents a fundamental structural switch that links presynaptic nanostructure to the earliest stages of synucleinopathy. A comparative summary of αSyn loss- and gain-of-function effects on presynaptic structure and physiology is provided in Table 1.

## 4. Synaptic Vesicle Structure Is Sensitive to Aging and α-Synuclein Perturbation

SVs are essential for precise neurotransmission, yet how their composition and architecture change with age or under conditions of αSyn dysregulation remains insufficiently defined [40]. This question has been examined using an integrative vesicle-profiling strategy applied to mouse models carrying either a genetic deletion of αSyn (αSynKO) or a modest, physiologically regulated overexpression of human αSyn (αSynBAC), neither of which fully recapitulates the temporal or pathological trajectory of human PD [11,41]. Employing a multimodal pipeline that combined cryo-electron tomography, quantitative proteomics, and lipidomics, these studies compared SV features across wild-type (WT), αSynKO, and αSynBAC mice at multiple ages, enabling parallel assessment of vesicle morphology, molecular composition, and lipid content under conditions of altered αSyn dosage. Importantly, these models interrogate physiological vulnerability and dosage sensitivity of αSyn function, rather than modeling late-stage loss of functional αSyn due to fibril sequestration. The analysis revealed pronounced age-dependent remodeling of SVs, intensified by either αSyn loss or overexpression [42,43]. In both models, presynaptic terminals exhibited reduced volume, smaller active zones (AZs), and fewer docked vesicles, indicative of impaired vesicle priming and neurotransmitter release [25]. Compositional alterations in vesicle-associated proteins and lipids accompanied these structural deficits [32]. However, such proteomic and lipidomic shifts represent associative molecular signatures that accompany altered αSyn dosage and aging, rather than independently proven drivers of synaptic impairment. Key age-dependent and genotype-specific proteomic and lipidomic signatures are summarized in Table 2.

In αSynKO mice, SVs were depleted of lipid raft-associated proteins and vesicle tethers, consistent with in vivo evidence that endogenous synuclein supports vesicle clustering and docking, as well as biochemical data showing facilitation of SNARE complex assembly via interaction with VAMP2 [15]. Conversely, αSynBAC mice displayed an accumulation of endocytic proteins and an enrichment of lipids that promote negative membrane curvature, suggesting altered vesicle trafficking dynamics and membrane stress under conditions of elevated αSyn expression, but not demonstrating the presence of Lewy pathology or dopaminergic degeneration. These observations support the idea that αSyn stabilizes SV integrity through lipid-protein interactions at the vesicle surface. Under physiological conditions, αSyn transitions between cytosolic and membrane-bound forms to scaffold vesicle membranes and maintain docking fidelity [53]. Disruption of this balance—either by absence or excess—has been causally linked in vivo to altered vesicle clustering and docking, whereas effects on SNARE assembly, membrane curvature, and endocytic recycling are supported primarily by ex vivo and biochemical evidence [8].

**Figure 1:**
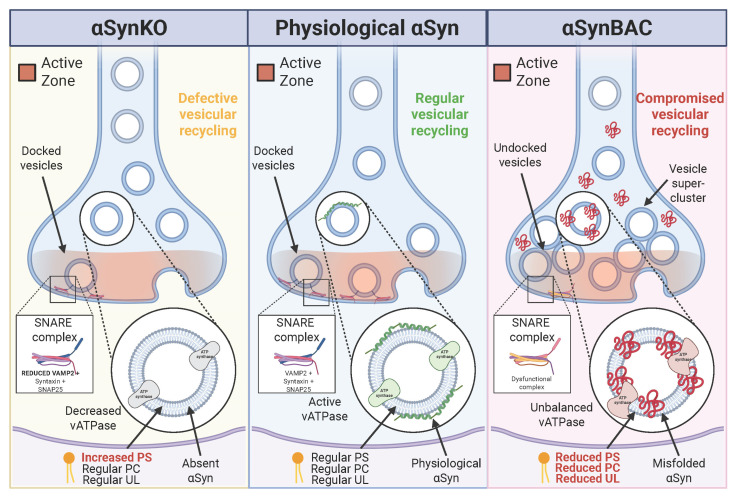
Conceptual model of α-synuclein-dependent synaptic vesicle dysfunction. α-Synuclein (αSyn) normally acts as a presynaptic, membrane-associated regulator of synaptic vesicle (SV) organization, supporting vesicle clustering, docking, SNARE assembly, and association with vesicle acidification machinery. In experimental PD-related models, αSyn loss-of-function (αSynKO) and gain-of-function (human αSyn expressed under endogenous control in αSynBAC mice) perturb distinct stages of the SV lifecycle without fully recapitulating human pathology. αSynKO models reveal physiological roles of αSyn, whereas αSynBAC synapses show age-dependent alterations in vesicle clustering, SNARE interactions associated with serine-129 phosphorylation, and vesicle lipid composition. Schematics depict proposed mechanistic relationships based on integrated in vivo and biochemical evidence rather than direct demonstrations of causality. The figure was generated with BioRender.com.

Notably, the distinct phenotypes in αSynKO and αSynBAC mice suggest that mechanistically divergent pathways converge on a broadly similar outcome: presynaptic dysfunction (Figure 1). Loss of αSyn reveals the dependence of vesicle organization on its physiological scaffolding functions, whereas excess αSyn perturbs membrane trafficking and curvature-sensitive processes. This dual vulnerability illustrates the dosage- and conformation-sensitive nature of the αSyn function.

Collectively, these findings show that SVs are dynamic substrates of age- and αSyn-dependent remodeling. They support the view that the presynaptic terminal represents an early site of vulnerability in PD, characterized by shifts in vesicle lipid–protein networks, yet these observations are insufficient to establish causality for neurodegeneration. Accordingly, therapeutic strategies aimed at preserving αSyn’s physiological interactions or stabilizing vesicle homeostasis should be regarded as exploratory approaches to modulate synaptic resilience and potentially influence early synaptic decline associated with aging and PD.

## 5. Aging and α-Synuclein Dosage Remodel Presynaptic Architecture

Neurons rely on the structural integrity of presynaptic terminals, where SVs cluster, dock, and fuse to enable efficient neurotransmitter release [40,54]. Although these terminals retain considerable plasticity, as evidenced by sustained synaptic remodeling and functional compensation during aging, the influence of α-synuclein levels on this ‘adaptive resilience’ remains unclear [4]. This question has been examined using αSynKO and αSynBAC mouse models [11].

Proteomic profiling of aged wild-type and αSynBAC mice revealed a coordinated upregulation of SV-associated proteins involved in tethering (the initial reversible attachment of a synaptic vesicle to the presynaptic active zone plasma membrane), fusion, and recycling [17]. These included ATP8A1, Bin1, Munc18-1, Rab27B, SV2B, synaptotagmins 1 and 2, syntaxin-1B, and synapsins, all essential for sustaining neurotransmission. This upregulation likely represents a compensatory adaptation to maintain synaptic function amid age-related stress [15,18]. Notably, ATP8A1 influences membrane curvature through phospholipid flipping, while Munc18-1 and syntaxins regulate SNARE complex formation and vesicle priming [55,56,57]. Evidence for their direct involvement in the structural phenotypes observed derives largely from prior biochemical and cellular studies rather than causal in vivo manipulation in these models.

Despite these molecular adaptations, structural analyses revealed consistent deficits in presynaptic morphology in both αSynKO and αSynBAC mice. Cryo-electron tomography and morphometric quantification showed reduced terminal volume, shortened AZs, and fewer docked vesicles than wild-type controls [11]. These alterations are directly demonstrated in vivo and establish a causal link between altered αSyn dosage and presynaptic architectural changes. Functionally, such changes are predicted to constrain synaptic efficacy, particularly during sustained or high-frequency activity, although direct physiological confirmation remains limited [58,59].

These findings expand the role of αSyn beyond vesicle binding, positioning it as a physiological organizer of presynaptic architecture that supports vesicle clustering and docking, with biochemical evidence supporting interactions with SNARE machinery through VAMP2 [15]. In αSynKO mice, reduced vesicle docking and impaired reserve pool mobilization suggest a breakdown in vesicle cycling. Conversely, prior studies of αSyn overexpression models reported pronounced disorganization of SV pools, vesicle mis-localization, and recycling deficits [60,61]. However, we should note that these phenotypes may stem from non-physiological expression levels, limiting their relevance to human disease.

**Figure 2:**
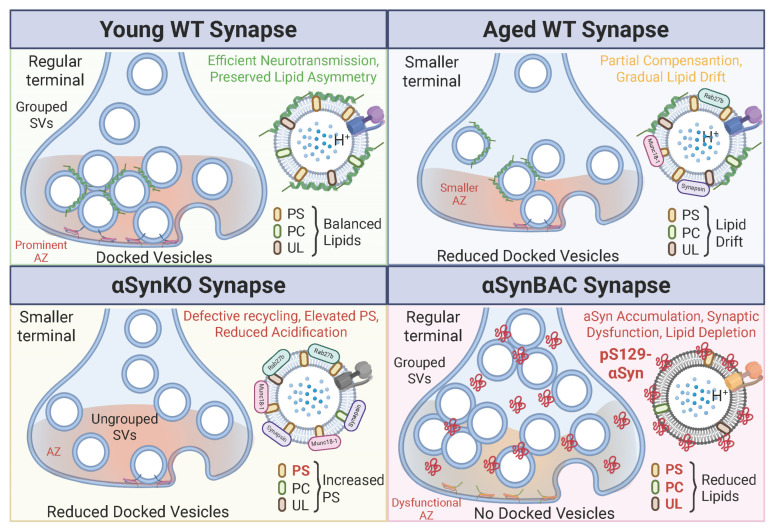
Proposed effects of aging and α-synuclein imbalance on synaptic vesicle architecture and presynaptic function. This schematic summarizes in vivo observations from mouse models integrated with biochemical and cell-based evidence across aging and α-synuclein (αSyn) perturbation. In young wild-type synapses, SVs form ordered clusters at the active zone, supported by αSyn-dependent tethering and vesicle acidification. Aging is associated with modest reductions in terminal size and vesicle docking, partially offset by compensatory upregulation of presynaptic proteins. αSynKO synapses, which reveal physiological roles of αSyn, show dispersed vesicle pools, altered vATPase subunits, and impaired recycling. αSynBAC synapses exhibit age-dependent changes, including phospho-S129 accumulation, altered vesicle clustering, and lipid remodeling. Schematics depict proposed mechanistic relationships rather than direct demonstrations of causality. The figure was generated with BioRender.com.

This issue was addressed using the αSynBAC model, which preserves native regulatory control of human αSyn expression [11]. This model revealed a subtler presynaptic remodeling phenotype: reduced AZ length and reduced vesicle docking, without overt neurodegeneration. These findings indicate that modest αSyn overexpression is sufficient to perturb presynaptic structure in vivo, but they do not demonstrate the emergence of Lewy pathology or dopaminergic cell loss [38]. These structural changes, though modest, imply a decline in synaptic resilience with age (Figure 2).

Together, these findings support an explanatory framework in which αSyn functions as a dosage-sensitive modulator of presynaptic health. Both loss and excess disrupt vesicle organization and SNARE-mediated fusion through distinct mechanisms, ultimately converging on impaired neurotransmission. These disruptions likely represent an early, potentially modifiable phase of PD pathogenesis. Understanding how αSyn regulates presynaptic architecture during aging may help identify therapeutic targets to stabilize synaptic integrity in prodromal PD.

## 6. Phosphorylation of α-Synuclein at ser129 Disrupts Vesicle Clustering in Aging Neurons

αSyn plays a pivotal role in maintaining presynaptic integrity by clustering SVs near AZs to support rapid neurotransmitter release. Direct in vivo evidence indicates that endogenous synuclein contributes to vesicle clustering and docking, whereas its facilitation of SNARE complex assembly is supported primarily by biochemical and cellular studies. This scaffolding function depends on its dynamic membrane association, where the protein adopts an α-helical conformation and promotes SNARE complex assembly [8,15]. However, its activity is sensitive to post-translational modifications that may alter its conformation, subcellular distribution, and interaction with vesicle membranes.

Among these modifications, phosphorylation at serine 129 (pS129) has emerged as a pathological hallmark of synucleinopathies [62,63]. Although this site is present at low levels under physiological conditions, it is highly enriched in Lewy bodies from PD brains [64,65,66]. The functional consequences of this modification remain debated, but several studies suggest that pS129 may impair αSyn’s native role and promote its pathogenic transformation [67,68,69]. This issue was examined in αSynBAC mice [11]. Immunohistochemical and biochemical analyses demonstrated an age-dependent accumulation of pS129 within presynaptic terminals in these mice. In contrast, wild-type mice expressing endogenous murine αSyn showed negligible pS129 levels, indicating a species-specific susceptibility to this phosphorylation state, potentially driven by sequence differences or differential regulatory mechanisms.

Mechanistic analyses showed that increased pS129 alters αSyn–vesicle interactions and is associated with abnormal vesicle clustering, rather than promoting vesicle mobility and efficient recruitment to release sites [63,66]. In vivo imaging and functional studies at vertebrate synapses demonstrate that excess pS129 induces vesicle declustering or immobilized subclusters, impairs vesicle recycling, and reduces the readily releasable pool [31]. Notably, these functional impairments occurred in the absence of overt neurodegeneration or visible protein aggregation, suggesting that pS129-driven alterations represent early, pre-aggregative pathological events in PD [70]. These results reveal a mechanism by which a single post-translational modification alters αSyn’s presynaptic function, promoting pathological effects on vesicle dynamics [65].

The αSynBAC model, by maintaining near-physiological expression levels, minimizes artifacts associated with protein overexpression and reveals early molecular events with high translational relevance, although it does not recapitulate Lewy pathology or dopaminergic degeneration [14,71]. Within this framework, pS129 may function as both a disease biomarker and an active modulator that disrupts synaptic vesicle dynamics and compromises synaptic homeostasis [7].

Collectively, these findings position pS129 as an important modifier of αSyn presynaptic function and a likely mediator of early synaptic dysfunction in PD. Future studies should identify the enzymes that regulate this modification in vivo and investigate whether and under what conditions pS129 facilitates later conformational transitions toward aggregation. Therapeutic strategies aimed at limiting αSyn phosphorylation or stabilizing its physiological multimeric state may help preserve vesicle mobility and delay the emergence of synaptic dysfunction in PD.

## 7. Species-Specific Sequence Modulates α-Synuclein Membrane Binding and Aggregation

Although human and mouse α-synuclein differ by only seven amino acids, these differences cluster in regions critical for membrane curvature sensing, lipid binding, and aggregation propensity. Biochemical and structural studies indicate that non-conserved residues can modulate αSyn biophysical properties, influencing membrane association, multimerization, and aggregation kinetics, with potential consequences for synaptic regulation [34,50].

Species-specific contributions were evaluated by comparing αSynBAC mice with wild-type mice expressing the murine αSyn ortholog [11]. Despite a shared genetic background, αSynBAC mice exhibited age-dependent synaptic alterations not observed in wild-type controls, including disrupted vesicle organization and enhanced phosphorylation at serine 129 [42,66]. These phenotypes are directly observed in vivo and implicate the human αSyn sequence as a contributing factor to altered presynaptic organization; however, they do not exclude interactions with age-dependent or cellular regulatory processes. The N-terminal domain (residues 1–60), essential for α-helical membrane binding and vesicle clustering, contains several amino acid substitutions between species [72,73]. Biophysical studies demonstrate that these differences influence αSyn’s affinity for curved membranes and its multimerization behavior, properties that are inferred to affect vesicle scaffolding at the active zone [74,75]. In αSynBAC mice, subtle disruptions in SV clustering were observed, accompanied by reduced vesicle docking, consistent with impaired presynaptic function.

The intrinsically disordered C-terminal domain (residues 96–140) regulates αSyn’s interaction with metal ions, chaperones, and post-translational machinery. Sequence variations in this region, particularly surrounding the conserved DPDNEA motif (residues 119–124), have been shown in biochemical and cellular systems to affect αSyn’s susceptibility to phosphorylation, nitration, and proteolytic cleavage [76,77,78]. These post-translational modifications are associated with altered aggregation kinetics and cellular toxicity, although their causal contribution to synaptic dysfunction in vivo remains incompletely resolved [64,65].

Notably, the human-specific DPDNEA motif appears to act as a conformational switch. Structural and in vitro aggregation studies indicate that even single-residue changes in this region can destabilize the native conformation, increasing β-sheet content and enhancing the nucleation of amyloid fibrils [75,79]. In αSynBAC mice, these sequence-dependent properties may contribute to age-related increases in pS129 and altered vesicle interactions, even in the absence of overt aggregation or Lewy pathology. Thus, species-specific sequence differences appear sufficient to bias membrane interactions and post-translational regulation, but not to induce fibrillization in isolation [80]. Therefore, despite equivalent genetic backgrounds, the divergent phenotypes between αSynBAC and αSynKO mice support the conclusion that the human αSyn protein confers unique biological properties relevant to PD [37].

From a translational perspective, these results highlight potential limitations of studying murine αSyn in isolation. Rodent models incorporating the human sequence could better replicate disease-relevant mechanisms, including early synaptic dysfunction and conformational vulnerability. Understanding how primary sequence determines αSyn’s structural transitions and functional roles is essential for developing targeted therapies that modulate its pathological states without disrupting its physiological scaffolding function.

## 8. α-Synuclein and Synapsins Regulate Vesicle Clustering via Dosage-Dependent Compensation

The precise spatial organization of SVs is crucial for maintaining synaptic efficacy and plasticity. This organization is maintained by a network of proteins that tether SVs to one another and to the cytoskeleton. Among these scaffolding proteins, synapsins and αSyn have emerged as central regulators of vesicle clustering. Although both contribute to presynaptic architecture, the degree to which they compensate for each other under physiological or pathological conditions remained unresolved [81,82].

Potential compensatory interactions were examined in vivo using αSynKO and αSynBAC mice [11]. Proteomic analyses revealed a reciprocal, dosage-dependent expression pattern. In αSynKO mice, synapsin levels were significantly elevated, consistent with a homeostatic response to preserve SV clustering. In contrast, synapsins were downregulated in αSynBAC mice, suggesting that human αSyn may suppress synapsin expression or stability through a feedback mechanism. In αSynKO mice, elevated synapsin failed to normalize presynaptic architecture: electron tomography showed smaller active zones and fewer docked vesicles than in wild-type controls [11], establishing that synapsin upregulation is insufficient to fully rescue vesicle docking and active zone organization in the absence of αSyn. Similarly, in αSynBAC mice, human αSyn failed to support normal vesicle tethering and appeared to override synapsins’ compensatory role, likely due to its propensity for misfolding or post-translational modification.

Biophysical studies have shown that both αSyn and synapsins can undergo liquid–liquid phase separation, forming dynamic condensates that organize SVs into functional clusters [83,84,85]. This property has been demonstrated in vitro and in cellular systems and provides a conceptual framework for understanding presynaptic organization, but direct visualization of such condensates in intact mammalian synapses remains limited. Within this framework, condensate formation is sensitive to the relative concentrations and conformational states of their components. In αSyn-deficient neurons, synapsin-rich condensates may maintain vesicle pools; however, under conditions of elevated or conformationally altered αSyn, biophysical studies predict destabilization of these assemblies, with potential consequences for vesicle mobility and recycling [14,86,87]. These effects are inferred from reconstituted systems and cellular models. Future studies combining live-cell biophysics and single-vesicle tracking in disease-relevant neuronal models will be required to directly test these predictions.

These findings define a dosage-sensitive regulatory axis between αSyn and synapsins that governs vesicle clustering and presynaptic organization. Disruption of this axis, whether through αSyn loss-of-function or toxic gain-of-function, is causally associated in vivo with altered vesicle docking and active zone structure, while accompanying proteomic changes likely reflect adaptive or maladaptive responses rather than primary drivers. Such perturbations may represent an early feature of synaptic vulnerability in PD [88]. These results highlight the importance of phase-separated protein assemblies in synaptic regulation and suggest that targeting condensate composition or enhancing compensatory mechanisms could offer novel therapeutic approaches to preserve synaptic resilience in neurodegenerative disease.

## 9. Presynaptic Regulators Link α-Synuclein Disruption to Early Synaptic Dysfunction

Efficient synaptic communication relies on tightly regulated protein–lipid interactions that govern SV trafficking, docking, and fusion. While αSyn has been widely studied for its role in PD, recent proteomic analyses have revealed previously unrecognized presynaptic proteins that respond to αSyn perturbation [89]. SV-enriched fractions from αSynBAC and αSynKO mice were profiled to identify early presynaptic regulators affected by αSyn imbalance [11].

A key finding was the dysregulation of ATP8A1, a P4-type ATPase that translocates phosphatidylserine and other aminophospholipids to the cytosolic leaflet of SV membranes [90]. In αSynBAC mice, ATP8A1 expression was altered compared to wild-type controls, an in vivo observation that associates increased αSyn dosage with changes in vesicle lipid-handling machinery. Since ATP8A1 governs the electrostatic surface properties of vesicles, essential for protein recruitment and membrane fusion, its dysregulation is inferred, based on prior biochemical and cellular studies, to influence SV biogenesis and docking efficiency [91]. These findings suggest that lipid microenvironment instability may be an early consequence of αSyn pathology [52].

The study also identified Rab27B as a candidate presynaptic target of αSyn imbalance. Rab27B, a small GTPase known for mediating vesicle tethering in secretory cells, was differentially expressed in both αSynBAC and αSynKO models. Although less characterized, its role in neurons is consistent with maintaining vesicle pool homeostasis during sustained synaptic activity [92,93]. Reduced Rab27B levels in αSynBAC mice therefore associate altered αSyn dosage with changes in vesicle positioning and priming capacity, possibly impairing synaptic responsiveness during high-frequency transmission.

Additional alterations were observed in terminal effectors of vesicle fusion. Both synaptotagmins 1 and 2, calcium sensors for SNARE-mediated exocytosis, and SV2B, a transmembrane glycoprotein regulating vesicle release probability, were downregulated in αSynBAC mice [11]. These proteins operate at the final step of the SV cycle, translating calcium influx into rapid neurotransmitter release [94]. Their coordinated downregulation represents an associative molecular signature of altered presynaptic state under increased αSyn dosage, suggesting potential constraints on the timing and fidelity of synaptic output.

A particularly striking result was the bidirectional regulation of CADPS2, a cytosolic priming factor essential for preparing SVs for calcium-triggered fusion. CADPS2 levels were reduced in αSynBAC mice but increased in αSynKO mice, revealing a dosage-sensitive regulatory relationship with αSyn [11]. This pattern suggests that αSyn modulates CADPS2 expression or function, possibly to balance vesicle priming capacity under varying synaptic loads. Previously implicated in dense-core vesicle release and neurodevelopmental disorders [95], CADPS2 may now be positioned as a convergence point between synaptic maintenance and PD-relevant presynaptic dysfunction [96].

**Figure 3:**
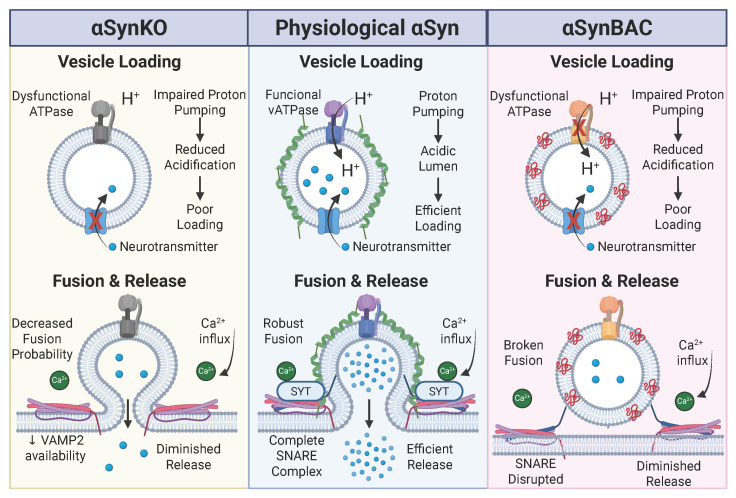
Proposed disruption of synaptic vesicle release by α-synuclein imbalance. α-Synuclein (αSyn) participates in multiple stages of the synaptic vesicle (SV) release cycle, including acidification, priming, fusion, and recycling. In vivo studies indicate that αSyn loss (αSynKO), which reveals its physiological roles, is associated with reduced vATPase subunits, decreased VAMP2 availability, and impaired SV acidification and recycling. In αSynBAC mice, age-dependent alterations include phospho-S129 accumulation, changes in SNARE-associated proteins, and lipid remodeling. Effects on fusion probability and membrane curvature are inferred from biochemical and cell-based studies. Both perturbations converge on impaired neurotransmitter release. Schematics depict proposed mechanisms rather than direct demonstrations of causality. The figure was generated with BioRender.com.

Collectively, these findings broaden the network of αSyn-sensitive presynaptic regulators beyond its classical SNARE interactions. Identifying ATP8A1, Rab27B, SV2B, synaptotagmins, and CADPS2 as responsive nodes supports a model in which αSyn imbalance is associated with coordinated alterations across multiple phases of the SV lifecycle, from membrane lipid handling to priming and fusion (Figure 3). The αSynBAC model, which preserves endogenous regulatory context and avoids overexpression artifacts, underscores how subtle, cumulative alterations in the presynaptic proteome precede neurodegeneration. These proteins may represent early biomarkers with mechanistic relevance for strategies aimed at preserving synaptic integrity in the prodromal (premotor) phase of PD.

## 10. α-Synuclein Imbalance Disrupts Vesicle Acidification via vATPase Dysregulation

Neurotransmitter loading into SVs depends on the generation of a transmembrane proton gradient, which acidifies the vesicular lumen and powers uptake by vesicular transporters. This gradient is driven by vacuolar-type H^+^-ATPases (vATPases), large multisubunit complexes composed of membrane-bound (V_0_) and cytosolic (V_1_) domains that cooperate to translocate protons into the vesicle interior [97,98]. Disruptions in vATPase function can impair vesicle acidification and diminish neurotransmitter storage, positioning these complexes as key regulators of synaptic efficacy, although direct measurements of vesicle pH are rarely obtained in vivo [39].

The influence of αSyn on vesicle acidification was assessed through quantitative proteomics of SV-enriched fractions from αSynKO and αSynBAC mice [11]. Both models exhibited a marked reduction in V_0_a1, the transmembrane subunit that forms the proton-conducting channel, an observation directly demonstrated in vivo at the protein level. This shared deficit indicates a convergent vulnerability in vesicle acidification pathways, suggesting that V_0_a1 expression or stability is sensitive to αSyn dosage, regardless of the direction of the effect [47,99].

Interestingly, the composition of other vATPase subunits diverged between models. In αSynBAC mice, expression of V_0_d1 and V_1_F1, components essential for ATP hydrolysis and rotor-stator coupling, was increased. In contrast, these subunits were reduced in αSynKO mice [47]. This bidirectional regulation indicates that αSyn dosage influences individual vATPase subunit levels and is likely to affect complex assembly or stoichiometry in a dosage-dependent manner. Based on established structure–function relationships of vATPase complexes, such changes are expected to alter the kinetic efficiency of proton pumping, with consequences for both the rate and capacity of SV acidification [16,100].

Although the precise contribution of each subunit to SV acidification remains unclear, the consistent depletion of V_0_a1 across both models likely represents a limiting step in maintaining vesicle proton gradients. The opposing patterns observed for V_0_d1 and V_1_F1 may reflect compensatory mechanisms in the presence of toxic αSyn species or destabilization of the complex in the absence of αSyn’s physiological scaffolding functions [5,101].

Together, these findings identify an association between αSyn imbalance and altered molecular composition of the vesicle acidification machinery. By modifying vATPase subunit levels and potentially complex assembly, αSyn perturbation—whether through absence or excess—is linked to changes in vesicle energetics, although direct causal effects on vesicle pH and neurotransmitter loading remain to be established. This convergence on vATPase regulation highlights vesicle acidification as a candidate early site of synaptic vulnerability in PD. Accordingly, targeting this axis may represent an opportunity to support synaptic efficiency at early stages.

## 11. Disrupted Endocytic Recycling Reveals a Synaptic Vulnerability Axis in α-Synuclein Models

Efficient SV recycling is essential for sustaining neurotransmission during prolonged neuronal activity. This process depends on a finely tuned molecular machinery that orchestrates membrane curvature, cargo selection, clathrin coat formation, and vesicle uncoating [45,54]. Disruption at any stage of this cycle can impair vesicle turnover, diminish neurotransmitter release, and promote synaptic fatigue [102].

Endocytic involvement was assessed using targeted proteomic analysis of SV-enriched fractions from αSynKO and αSynBAC mice [11]. Both models exhibited selective alterations in key regulators of vesicle retrieval and membrane remodeling, identifying the endocytic axis as a common site of vulnerability [44,103]. One prominent example was AP1B1, a subunit of the adaptor protein complex involved in cargo recognition during clathrin-mediated endocytosis. In both αSynKO and αSynBAC mice, AP1B1 levels were reduced, suggesting that αSyn imbalance interferes with cargo sorting and SV identity [104]. This was accompanied by decreased expression of synaptojanin 1 (SYNJ1), a lipid phosphatase essential for PI(4,5)P_2_ turnover and vesicle uncoating. Given that SYNJ1 mutations are associated with juvenile Parkinsonism [46], its dysregulation in αSyn models provides a mechanistic link between genetic and sporadic forms of the disease.

Additional deficits were observed in BIN1 and PICALM, two proteins that coordinate membrane curvature and clathrin-mediated budding. BIN1 functions at the interface of membrane bending and actin remodeling, while PICALM helps recruit clathrin and adaptors to form vesicles [105]. Both proteins were reduced in αSynBAC mice, linking increased αSyn dosage to changes in endocytic machinery composition at the presynaptic terminal. Notably, BIN1 and PICALM have also been genetically linked to Alzheimer’s disease and other neurodevelopmental disorders, suggesting a shared vulnerability of vesicle trafficking pathways across neurodegenerative conditions.

These alterations extend beyond simple vesicle depletion. Based on established roles of these proteins, impaired recycling is inferred to influence SV maturation, neurotransmitter refilling, and release efficiency, although these functional consequences were not directly measured in vivo in the αSyn models discussed [14,23]. Whether these changes reflect direct effects of altered αSyn dosage or compensatory responses to perturbed vesicle dynamics remains unresolved. However, the consistent changes across models support a causal association between αSyn dosage imbalance and the integrity of the vesicle recycling machinery [60].

Collectively, these findings identify endocytic recycling as a recurrent presynaptic vulnerability axis associated with αSyn imbalance [101]. By associating altered αSyn dosage with changes in vesicle retrieval and renewal machinery, these data suggest mechanisms through which synaptic resilience may be reduced at early stages, potentially contributing to functional decline. This perspective emphasizes the importance of considering the full SV lifecycle, including retrieval and refilling, when evaluating therapeutic strategies aimed at supporting synaptic function in prodromal PD and related synucleinopathies.

## 12. Vesicle Lipid Loss Precedes Synaptic Dysfunction and α-Synuclein Aggregation

SV membranes are highly specialized structures whose lipid composition governs their morphology, dynamics, and interactions with presynaptic proteins. Lipids such as phosphatidylserine (PS) and lysophosphatidylcholine (lysoPC) are essential not only for maintaining vesicle curvature and charge but also for stabilizing membrane-bound conformations of αSyn, a presynaptic protein implicated in PD [1,106]. Disruption of this lipid–protein interface has therefore been proposed, based largely on biochemical and cellular studies, as a potential contributor to synaptic vulnerability in synucleinopathies [32].

Lipidomic profiling of purified SVs from αSynBAC mice revealed early and progressive remodeling of vesicle lipid composition in vivo [11]. Compared to wild-type controls, αSynBAC mice exhibited a progressive, age-dependent decline in total SV lipid content. This reduction emerged before overt neurodegeneration, suggesting that αSyn expression alters lipid homeostasis independently of terminal pathology.

Detailed profiling revealed specific depletion of PS and lysoPC, lipid species critical for anchoring αSyn’s amphipathic N-terminal helix to the vesicle surface. Biophysical and structural studies indicate that these acidic, cone-shaped lipids promote the α-helical multimer formation of αSyn at the membrane, a conformation necessary for vesicle clustering and SNARE complex assembly [107,108,109,110]. In the αSynBAC model, loss of these lipids is associated with reduced membrane engagement of αSyn; however, the consequent shift toward cytosolic or aggregation-prone species is inferred from known structure–function relationships rather than directly demonstrated in vivo [111]. This lipid deficiency may initiate a structural transition predisposing αSyn to pathogenic assembly.

Crucially, these findings shift vesicle lipid dysregulation from being viewed solely as a downstream consequence of neurodegeneration to an early and contributory factor in αSyn pathology [32,112,113]. The data suggest a feedforward mechanism: declining lipid content weakens αSyn–membrane interactions, increasing cytosolic αSyn, which may further destabilize vesicle architecture, although this sequence remains inferential [21,114,115]. This cycle could underlie the gradual presynaptic decline observed in prodromal PD and escape detection in conventional post-mortem analyses focused on late-stage pathology [7,116].

Collectively, these results broaden the pathogenic framework of αSyn-related disorders to include lipid-mediated vesicle destabilization as an early trigger. They highlight SV lipid loss as a mechanistic bridge between αSyn biology and synaptic vulnerability. Therapeutic strategies that preserve vesicle lipid composition, or restore the lipid environment required to maintain αSyn in its functional, membrane-bound form, may offer a novel approach to delaying αSyn aggregation and the onset of synaptic degeneration in PD.

## 13. α-Synuclein Remodels Vesicle Lipid Composition and Modulates Membrane Biophysics

αSyn is well known for its ability to bind acidic phospholipids. However, emerging evidence suggests it also actively regulates the lipid architecture of presynaptic membranes. This dual role, as both lipid sensor and effector, positions αSyn at the intersection of membrane biophysics and synaptic function. In PD, where αSyn misfolding is tightly linked to synaptic dysfunction, its influence on vesicle lipid composition remains incompletely defined [49].

The influence of αSyn on vesicle lipid composition was investigated through lipidomic profiling of purified SVs from αSynBAC mice, which express human αSyn under endogenous regulatory control [11]. Relative to wild-type controls, these vesicles displayed selective alterations, including reduced phosphatidylcholine (PC) and enrichment of phosphatidylserine (PS) and lysophospholipids [117]. These changes were detected in vivo prior to overt neurodegeneration, and partially overlap with lipid signatures reported in post-mortem PD brain tissue, suggesting that disease-relevant lipid imbalances can emerge early but may evolve further with disease progression [118,119].

Alterations in lipid class composition are expected, based on established membrane biophysics, to affect the physical properties of SV membranes. PC maintains bilayer integrity and flexibility, while PS and lysophospholipids introduce negative charge and curvature stress—features that can influence vesicle formation, docking, and fusion [48,51,55]. Reduced PC is inferred to constrain bilayer fluidity and deformability, impairing vesicle mechanics. Concurrently, PS enrichment is expected to enhance αSyn’s membrane affinity, potentially stabilizing disordered conformers prone to oligomerization [120,121]. Together, these lipid shifts are likely to exacerbate SV instability and bias αSyn toward aggregation-prone conformational states.

Mechanistically, αSyn has been shown in cellular and biochemical systems to modulate phospholipase activity and localize to mitochondria–endoplasmic reticulum contact sites, key zones for lipid biosynthesis and exchange [122,123]. The lipid alterations observed in αSynBAC mice are therefore consistent with a model in which αSyn participates in lipid regulatory networks, although direct causality between αSyn activity and specific lipid changes has not been demonstrated in vivo. A feedforward interaction is suggested, whereby αSyn-associated lipid remodeling may increase susceptibility to αSyn mislocalization and synaptic stress, which could further destabilize vesicle organization and neurotransmission [11,84,113].

These findings broaden the conceptual framework of αSyn biology by implicating its role in the dynamic regulation of vesicle lipid composition [32,49]. Rather than indicating that lipid dysregulation is a sole driver of pathology, the data support a model in which altered membrane biophysics acts as an early modifier of synaptic vulnerability that intersects with αSyn dosage and conformational state [124]. Accordingly, SV lipid homeostasis emerges as a potentially modifiable aspect of synaptic resilience in synucleinopathies. Interventions aimed at supporting balanced lipid environments—such as maintaining appropriate PC and PS levels—may help stabilize physiological αSyn–membrane interactions and mitigate progression toward synaptic dysfunction.

## 14. Dual α-Synuclein Dysfunctions Converge on Synaptic Vesicle Failure

Although αSyn pathology in PD has traditionally been attributed to toxic protein aggregation, accumulating evidence indicates that disruption of αSyn’s physiological presynaptic functions can also contribute to synaptic dysfunction. This dual perspective frames αSyn not only as a source of toxic species but also as a dosage- and context-sensitive regulator of SV dynamics whose loss of function may render presynaptic terminals vulnerable [6].

This hypothesis was evaluated using a vesicle-omics approach comparing two genetically distinct models: αSynBAC mice, which express human αSyn at near-physiological levels, and αSynKO mice lacking endogenous αSyn [11]. Despite opposing effects on αSyn dosage, both models exhibited in vivo alterations in synaptic vesicle organization. In αSynBAC mice, age-dependent impairments in vesicle docking, neurotransmitter release, and recycling were consistent with toxic gain-of-function [60]. In contrast, αSynKO mice displayed more severe deficits, including reduced vesicle clustering, distorted membrane curvature, and presynaptic terminal shrinkage, indicating that the loss of αSyn’s normal function compromises synaptic architecture.

Mechanistically, the two models revealed distinct but convergent routes to synaptic destabilization. αSynBAC mice exhibited aberrant SNARE complex formation and disrupted protein–lipid interactions, implicating misfolded or excess αSyn in vesicle trafficking dysfunction [89]. Meanwhile, αSynKO mice lacked membrane scaffolding and curvature-sensing capacity typically mediated by αSyn’s N-terminal helix, resulting in fragmented vesicle pools and impaired synapsin-dependent condensate formation. The profound structural collapse observed in αSynKO terminals suggests that αSyn is not merely accessory but central to presynaptic architecture [25,38].

These observations are consistent with observations in both human and experimental models. In PD brains, soluble αSyn is significantly depleted in vulnerable regions before neuronal loss is detected [125,126]; however, these postmortem findings predominantly reflect late-stage disease and do not establish when such depletion first emerges in the clinical course. Likewise, seeded aggregation studies show that pathogenic αSyn fibrils can sequester functional αSyn into insoluble complexes, effectively depleting its active pool without overt toxicity in cellular and experimental models [127,128]. At present, the primary significance of these observations lies in their mechanistic implications for synaptic vulnerability rather than in their immediate suitability as early diagnostic biomarkers in living patients. While these observations suggest convergence on reduced availability of functional αSyn, they do not establish equivalence between genetic deletion and aggregation-mediated sequestration.

Together, these findings suggest a common endpoint: the loss of functional αSyn, regardless of whether the initiating event is overexpression or depletion. These data support a revised model of PD pathogenesis in which synaptic failure results from the combined effects of αSyn toxic gain-of-function and physiological loss-of-function [7]. This paradigm shift has clear therapeutic implications. Strategies focused solely on αSyn clearance may inadvertently exacerbate synaptic dysfunction by further depleting the functional pool. A more effective approach may involve preserving αSyn’s membrane-bound, multimeric, and non-aggregated physiological state, stabilizing vesicle dynamics, and protecting synaptic integrity in the early stages of the disease.

## 15. Synaptic Vesicle Disruption Emerges as an Early Therapeutic Target in Parkinson’s Disease

Traditional therapeutic approaches for PD have primarily focused on dopamine replacement and the treatment of late-stage neurodegeneration [129]. Nevertheless, recent evidence points to presynaptic dysfunction, particularly at SVs, as an early, potentially initiating event in PD pathogenesis. This evolving view reframes αSyn not only as a pathological aggregate but also as a presynaptic scaffold essential for vesicle organization, membrane curvature sensing, and neurotransmitter release [11].

Early vulnerability was assessed by profiling SV composition in αSynBAC mice expressing human αSyn at near-physiological levels [11]. Lipidomic and proteomic analyses revealed targeted disruptions in SV architecture that preceded neurodegeneration [32]. Loss of PC and enrichment of PS, lipids that regulate membrane curvature and fluidity, were observed [117,130]. Biophysical studies suggest that such lipid shifts can bias αSyn toward increased membrane association and altered conformational ensembles; however, enhanced aggregation propensity is inferred from structure–function relationships rather than directly demonstrated in these models [119,131]. Alongside lipid alterations, key SV-associated proteins exhibited dosage- and conformation-dependent responses to αSyn imbalance [104]. Regulators of the vesicle lifecycle, such as synapsins (clustering), CADPS2 (priming), Rab27B (trafficking), and vacuolar H^+^-ATPase subunits (acidification), showed expression changes sensitive to αSyn dosage and conformation [11]. These findings suggest a coordinated response to presynaptic stress, linking protein and lipid dynamics in the early stages of synaptic dysfunction.

Therapeutically, these findings support the use of dual intervention strategies. First, stabilizing αSyn in its native, membrane-bound multimeric form may preserve its physiological function and prevent toxic aggregation [15,108,120,132]. Second, restoration of SV lipid composition, through modulation of lipid metabolic enzymes or targeted lipid supplementation, may help reconstitute a biophysical environment that supports healthy αSyn–membrane interactions.

Importantly, these molecular changes offer potential biomarkers for early PD detection. Altered expression of CADPS2 or deviations in SV lipid ratios, detectable in extracellular vesicles or cerebrospinal fluid, may reflect presynaptic health before clinical symptoms emerge [133,134]. Such biomarkers may aid in stratifying risk or monitoring early disease processes and enable therapeutic intervention during the prodromal phase of PD, shifting treatment paradigms from symptomatic management toward disease modification [135,136].

Together, these results position the synaptic vesicle as a central node of vulnerability and opportunity in PD. Targeting the αSyn–SV interface may help stabilize presynaptic integrity, delay neurodegenerative progression, and redefine therapeutic timing. Intervening at this early synaptic stage, before irreversible dopaminergic neuron loss, offers a promising strategy to reshape the course of PD.

## 16. Drug Discovery: Targeting the α-Synuclein–Vesicle Interface in Parkinson’s Disease

Recent findings have expanded our understanding of PD, reframing it as a disorder in which SV dysfunction driven by αSyn imbalance may play a central role. This pathophysiological axis involves disrupted protein–lipid interactions, altered vesicle architecture, impaired SNARE complex formation, and defective acidification, many of which are observed in vivo before overt neurodegeneration in experimental models [7]. These early molecular changes offer a unique opportunity to develop disease-modifying therapies (Table 3). This section identifies three high-priority molecular targets: vATPase subunits, SNARE complex components (especially VAMP2), and vesicle lipid composition (particularly phosphatidylserine and phosphatidylcholine), which emerge as sensitive nodes within αSyn-associated SV pathology [11,15,108,119].

### 16.1. Targeting vATPase to Restore Vesicle Acidification

The acidification of SVs is essential for neurotransmitter loading and synaptic fidelity. This process depends on vacuolar H^+^-ATPases (vATPases), which generate a proton gradient across the vesicle membrane [97]. Proteomic analyses of SV-enriched fractions from αSynKO and αSynBAC mice revealed reduced abundance of the vATPase V_0_a1 subunit, alongside divergent regulation of V_0_d1 and V_1_F1, establishing an in vivo association between altered αSyn dosage and vATPase subunit composition [11].

Loss of acidification is inferred to compromise vesicle loading (Figure 3), impair neurotransmission, potentially contributing to presynaptic vulnerability. Given the convergence of αSyn loss- and gain-of-function on vATPase dysfunction, pharmacological strategies that stabilize or enhance vATPase activity, particularly V_0_a1 expression or function, may help maintain vesicle energetics. vATPase modulators have shown promise in lysosomal disorders and cancer [16,100], but specificity remains a challenge due to the complex subunit composition and widespread distribution of vATPase isoforms. Selective activation of synapse-enriched vATPase variants or protection of αSyn–vATPase interactions could represent viable strategies in PD.

### 16.2. Stabilizing SNARE Complex Assembly via VAMP2 Protection

Efficient vesicle fusion depends on the formation of a ternary SNARE complex composed of synaptobrevin/VAMP2, syntaxin-1, and SNAP25. αSyn interacts directly with VAMP2 to facilitate SNARE complex formation, a role supported by biochemical and cellular studies and indirectly reflected in vivo by altered SNARE-associated protein profiles. In αSynKO mice, loss of αSyn scaffolding is associated with reduced vesicle docking, while in αSynBAC mice, conformationally altered αSyn, often accompanied by increased pS129, correlates with disrupted SNARE organization [11,108]. Disruption of this complex impairs calcium-triggered exocytosis and diminishes neurotransmitter release (Figure 3).

Pharmacological stabilization of SNARE proteins, especially VAMP2, represents a promising therapeutic approach. Small molecules or peptides that mimic αSyn’s physiological binding to VAMP2 could help preserve SNARE assembly in settings of αSyn depletion or sequestration, although such approaches remain untested in vivo. Alternatively, blocking pS129 phosphorylation [64,65] could preserve αSyn’s functional conformation, potentially preserving VAMP2 interaction. Kinase inhibitors targeting enzymes responsible for S129 phosphorylation (e.g., PLK2, CK1) are under investigation in neurodegenerative models and may be repurposed for synaptic protection [67,68].

### 16.3. Restoring Vesicle Lipid Composition to Preserve αsyn Function

SV lipid composition is a critical determinant of vesicle curvature, charge, and protein binding. Phosphatidylserine (PS) and phosphatidylcholine (PC) are especially important for αSyn membrane anchoring and function [1,108]. Lipidomic analyses of αSynBAC mice revealed progressive depletion of PC and PS, alongside enrichment in curvature-inducing lipids, destabilizing αSyn’s membrane-bound multimeric state and promoting misfolding [11]. This lipid loss precedes protein aggregation and synaptic decline (Figure 1 and Figure 2), positioning vesicle lipid metabolism as a prime therapeutic target.

Drug discovery efforts may leverage two complementary strategies: (1) restoration of lipid biosynthesis through modulation of phosphatidylserine synthases or PC biosynthetic pathways, and (2) targeted lipid delivery using liposome-based nanocarriers or blood–brain barrier-permeable lipid precursors [48,112]. Preclinical studies in Alzheimer’s disease models have shown that lipid supplementation can normalize membrane homeostasis and improve cognitive performance [118]. Whether similar strategies can meaningfully influence PD-related synaptic dysfunction remains to be determined.

An alternative strategy is to modulate lipid–protein interactions directly. Small molecules or peptides that stabilize αSyn’s α-helical conformation at membranes may counteract its cytosolic misfolding and aggregation [22,113]. Screening platforms that assess αSyn–lipid binding dynamics in vitro and iPSC-derived neurons may aid in identifying such stabilizers.

However, pharmacological manipulation of vesicle lipid composition or stabilization of membrane-bound αSyn multimers presents significant challenges. Excessive or poorly controlled stabilization of αSyn–membrane interactions could inadvertently favor aberrant self-association or perturb vesicle lipid organization, with unintended consequences for synaptic function [139,140,141]. Moreover, lipid-targeted interventions must contend with delivery, specificity, and systemic metabolic effects [138]. These considerations underscore that therapeutic strategies aimed at modulating αSyn–lipid interactions should prioritize finely tuned, context-dependent modulation rather than rigid stabilization [141].

### 16.4. Translational Outlook

Therapies aimed at preserving synaptic function in PD must target the earliest molecular events in disease progression. Unlike traditional approaches focused on αSyn clearance, the interventions described here aim to stabilize αSyn’s physiological roles and restore lipid–protein balance within vesicles, thereby preserving neurotransmission before neurodegeneration becomes irreversible.

By focusing on three interrelated targets, vATPase, VAMP2, and SV lipid composition, this drug discovery strategy provides a mechanistic, synapse-centered framework aimed to early disease modification. Future screens should incorporate phenotypic assays that measure vesicle clustering, acidification, and exocytosis in live neurons. Human induced pluripotent stem cells (iPSCs), patient-derived adult cells reprogrammed to an embryonic-like pluripotent state and differentiated into disease-relevant neurons, preserve individual genetic risk. Combining these iPSC models with lipidomic and proteomic profiling will directly connect molecular alterations to functional and therapeutic outcomes.

Importantly, many of the therapeutic strategies discussed here remain conceptual and face substantial translational barriers. vATPase modulation is complicated by its ubiquitous expression and essential roles in vesicular and lysosomal homeostasis [16,142], SNARE complexes present intrinsic challenges for pharmacological targeting due to their tight protein–protein interfaces [143,144], and lipid-based interventions raise concerns regarding brain delivery, compartmental specificity, and systemic metabolic effects [145,146]. Moreover, achieving selective modulation at presynaptic terminals without disrupting global vesicle trafficking or lysosomal function remains a major challenge [147]. Ultimately, validation of these mechanisms and therapeutic targets will require longitudinal clinical studies, human biomarker development, and interventional trials, as preclinical models alone cannot establish clinical efficacy. As such, these approaches should be viewed primarily as frameworks to guide target validation and early-stage screening, rather than as near-term pharmacological solutions.

## 17. Discussion

Here, we support a reframing of PD as a disorder in which SV dysfunction represents an early and convergent pathogenic axis, rooted in altered physiological roles of α-synuclein. By integrating proteomic, lipidomic, and ultrastructural data from αSynKO and αSynBAC mice, we argue that vesicle deregulation precedes neurodegeneration and is driven by both the loss and pathological accumulation of α-synuclein.

Importantly, functional and electrophysiological studies from multiple model systems provide independent evidence that presynaptic impairment emerges before overt neurodegeneration, complementing the structural and molecular findings discussed in this review. Human iPSC-derived neurons carrying PD-associated mutations exhibit reduced synaptic responsiveness and decreased synapse number, despite the absence of neurodegeneration, indicating that synaptic impairment can emerge as an early intrinsic feature of the disease trajectory [148]. In vivo analyses in rodent models expressing pathogenic α-synuclein variants reveal early deficits in neurotransmission, synaptic energy failure, impaired plasticity, and the accumulation of dysfunctional organelles at dopaminergic terminals prior to the appearance of motor symptoms or dopaminergic cell loss [149].

Invertebrate models similarly display presynaptic electrophysiological defects and loss of synaptic protein expression that precede neuron loss, supporting an evolutionary conserved sequence in which synaptic dysfunction arises upstream of cell loss [150]. Causal links between α-synuclein perturbation and presynaptic failure are further supported by acute manipulation experiments, in which loading monomeric α-synuclein into intact mammalian synapses rapidly inhibits vesicle endocytosis and reduces transmission fidelity during high-frequency stimulation, while leaving basal release largely preserved [151]. Additional rodent studies report early abnormalities in vesicle cycling, short-term plasticity, and exo-endocytic coupling prior to structural degeneration of the nigrostriatal pathway [152,153].

Human imaging studies using PET and post-mortem analyses further indicate that reductions in presynaptic terminal markers, including SV2A, are detectable at early disease stages and may precede measurable neuronal loss in the substantia nigra [101,154,155]. Mechanistic insights from electrophysiological and functional assays indicate that α-synuclein accumulation perturbs synaptic vesicle trafficking, SNARE complex efficiency, and neurotransmitter release kinetics, often in the absence of overt aggregation or degeneration [7,151,156,157]. Consistent with this framework, mutations in endocytic and trafficking regulators, including VPS35 and auxilin, produce similarly early defects in synaptic transmission demonstrable by both functional and morphological approaches [148,158,159]. Collectively, these findings provide convergent functional evidence that presynaptic impairment represents an early and biologically meaningful event in Parkinson’s disease, preceding neuronal loss and plausibly contributing to the subsequent neurodegenerative cascade, even though the extent to which synaptic failure alone is sufficient to drive degeneration remains to be fully established.

Across this spectrum, three recurrent molecular vulnerabilities can be identified: impaired vesicle acidification due to dysfunctional vATPase complexes, destabilization of SNARE assembly secondary to VAMP2 depletion, and progressive loss of vesicular phospholipids that disrupt membrane curvature and α-synuclein anchoring. These findings highlight tractable entry points for early therapeutic intervention and present a rationale for synapse-centered disease modification. However, the advancement of this research agenda is hindered by a series of conceptual and methodological challenges that must be addressed to enable clinical translation.

First, limited regional resolution in most studies obscures nucleus-specific vesicle abnormalities that are likely relevant to early PD pathogenesis. Analyses performed on whole-brain or bulk synaptosomal fractions dilute critical changes occurring in regions such as the substantia nigra pars compacta, where dopaminergic neurons possess unique lipid and vesicle signatures [160].

Second, cell-type specificity remains insufficiently resolved. Neuronal diversity is associated with considerable heterogeneity in synaptic vesicle composition, release kinetics, and α-synuclein function, yet most studies fail to distinguish between dopaminergic, glutamatergic, and GABAergic populations [17,161]. Advances in cell-specific labeling, sorting, and lipid profiling are necessary to overcome this limitation.

Third, the predominantly cross-sectional nature of existing studies limits insights into temporal progression of vesicle dysfunction. The current results suggest a sequence in which initial lipid drift and VAMP2 redistribution transition toward more entrenched synaptic failure, yet these trajectories cannot be resolved without longitudinal and inducible models [8,137].

Fourth, membrane leaflet asymmetry, a key determinant of α-synuclein binding to negatively charged inner-membrane lipids such as phosphatidylserine, is routinely disrupted by standard lipidomic techniques, precluding the characterization of this essential biophysical property in disease [117,136].

Fifth, poor characterization of vesicle lipid nanodomains within the vesicle membrane hinders the analysis of functional microdomains where SNARE assembly, α-synuclein anchoring, and calcium-sensing proteins converge. These structures are too small and dynamic to isolate with conventional biochemical or imaging tools, and yet they may represent the most vulnerable subcompartments in PD [8].

Sixth, the limited capacity to resolve vesicle pools within synapses obscures functional distinctions between the readily releasable, recycling, and reserve vesicle populations. The observed reduction in docked vesicles in αSynBAC mice, for example, likely reflects the selective vulnerability of the fusion-competent pool, a finding that would be missed in bulk analysis [14,18,110].

Seventh, the underrepresentation of sex-based differences in mechanistic studies weakens the generalizability of current findings. Differences in vesicle turnover, lipid metabolism, and hormonal regulation may influence α-synuclein biology and PD susceptibility but remain largely unexplored in preclinical models. Both biological divergence and limited human validation constrain translational generalization [162].

Eighth, while human iPSC-derived dopaminergic neurons offer an accessible platform, their synaptic architecture and vesicle profiles may not recapitulate those of mature substantia nigra neurons. Complementary studies in post-mortem brain tissue and refined humanized mouse models are needed to verify the relevance of vesicle-based targets [160].

Ninth, a consistent lack of molecular–functional correlation in existing literature limits our ability to attribute synaptic phenotypes to specific lipid or protein changes. The association between vATPase downregulation and reduced vesicle acidification, for instance, implies impaired neurotransmitter loading, but direct electrophysiological validation is lacking. Integrative approaches combining molecular profiling with functional readouts, such as synaptic release probability or vesicle fusion dynamics, will be critical [47,163].

Tenth, the distinction between pathological drivers and compensatory responses remains difficult to establish. Lipid depletion, VAMP2 loss, or changes in SNARE configuration may reflect protective adaptations rather than direct toxic mechanisms. Only interventional studies that restore specific molecular elements and measure their effects on synaptic function can clarify their role in disease progression [164,165].

Eleventh, the integration of α-synuclein-dependent synaptic vesicle dysfunction with other early pathogenic stressors remains incomplete. Early Parkinson’s disease is also characterized by mitochondrial Ca^2+^ handling defects, oxidative metabolic stress, and ER–Golgi trafficking perturbations, particularly in dopaminergic neurons with high energetic demand [166,167,168]. These processes can directly influence vesicle acidification, lipid composition, and endocytic capacity [46,169], supporting the view that synaptic vesicle failure represents a convergent pathogenic node rather than a singular initiating mechanism [5].

These limitations notwithstanding, the identification of converging vesicular deficits in both αSynKO and αSynBAC states supports a mechanistically coherent approach to drug discovery. First, therapies that stabilize vATPase function may restore vesicle acidification and support neurotransmitter loading. The V_0_a1 subunit, selectively downregulated in α-synuclein-altered states, represents a promising target for molecular rescue [11,16]. Second, protecting VAMP2 from depletion or aggregation, either by stabilizing SNARE interactions or preventing α-synuclein phosphorylation at serine-129, may preserve vesicle docking and fusion capacity [65,67,108]. Third, replenishing depleted phosphatidylserine and phosphatidylcholine pools may prevent α-synuclein detachment from membranes and mitigate toxic aggregation [1,118]. Such interventions could include lipid precursors, membrane-targeted nanocarriers, or small molecules that stabilize the helical conformation of α-synuclein at the vesicle surface.

Crucially, these strategies are designed not to eliminate α-synuclein but to restore its physiological function as a synaptic organizer, bridging the vesicle lipidome with the exocytic machinery. By targeting the earliest nodes of dysfunction, vesicle acidification, membrane anchoring, and SNARE assembly, before irreversible degeneration occurs, this approach offers the possibility of actual disease modification. A renewed focus on the synapse, supported by advances in lipidomic resolution, spatial proteomics, and functional validation, may ultimately yield the next generation of PD therapies.

## Figures and Tables

**Table 1:** Dual impact of α-synuclein dysfunction on synaptic vesicle architecture and presynaptic physiology.

Presynaptic Feature	Loss-of-Function (αSynKO)	Gain-of-Function (αSynBAC)
SV terminal morphology [11,38]	Reduced terminal size and AZ length	Smaller terminals with AZ shortening
SV docking [11,25]	Fewer docked SVs; impaired tethering to AZ	Fewer docked SVs despite increased clustering
SV clustering [23,25]	Increased SV density in distal pools; impaired spatial segregation	Elevated SV clustering at 10 months; tighter inter-vesicle spacing
AZ organization [11,38]	Shortened AZ; disorganized vesicle distribution	AZ clustering was preserved, but the spatial organization was disrupted
SV recycling pool [14,33]	Increased free (unclustered) SVs in sucrose gradients	There is no change in free SVs despite the ultrastructural increase in clustering
SNARE complex integrity [8,15]	Reduced VAMP2; decreased SNARE assembly	Disrupted VAMP2–syntaxin interactions via pS129 αSyn; SNARE uncoupling
SV acidification [11,39]	Downregulated vATPase subunits (V0a1, V0d1, V1F1)	Selective vATPase subunit dysregulation (↑ V0d1, V1F1; ↓ V0a1)

SV: synaptic vesicle; AZ: active zone; αSynKO: α-synuclein knockout mouse; αSynBAC: human α-synuclein BAC transgenic mouse; SNARE: soluble NSF attachment protein receptor; VAMP2: vesicle-associated membrane protein 2; vATPase: vacuolar-type H^+^-ATPase; ↑: increased; ↓: decreased. References provide representative support for the listed presynaptic effects of α-synuclein gain- and loss-of-function; mechanistic interpretation and model-specific limitations are discussed in the main text.

**Table 2:** Age- and genotype-specific alterations in synaptic vesicle proteome and lipidome.

Molecular Category	Age-Related Changes (WT)	αSynKO-Specific Changes	αSynBAC-Specific Changes
SV-associated proteins [18,44]	↑ ATP8A1, Rab27B, SV2B, Synaptotagmins 1/2, Syntaxin-1B, Munc18-1	↑ CADPS2, Synapsins 1/2, NSF; ↓ vATPase (V0a1, V0d1, V1F1)	↓ CADPS2, Synapsins 1/2/3, AP1B1, PICALM; ↑ vATPase (V0d1, V1F1); ↓ V0a1
Endocytic regulators [45,46]	↑ BIN1	↑ Synaptojanin-1, AP1B1	↓ PICALM; ↑ BIN1
SNARE machinery [15,40]	↑ Munc18-1, Syntaxin-1B	Altered SNARE partner dynamics	Impaired SNARE interactions via pS129 αSyn phosphorylation
Acidification machinery [39,47]	Subtle vATPase shifts	↓ V0a1, V0d1, V1F1	↑ V0d1, V1F1; ↓ V0a1
Lipid classes (mass spec) [48,49]	Stable PC, PE, PS, CHOL, lyso-lipids	↑ PS at 10 months; CHOL unchanged	↓ PC, PE, CHOL, PS, lysoPC, lysoPE, lysoPS
Lipid saturation [50,51]	Stable mono-/polyunsaturated profile	Saturation unchanged	↓ unsaturated species; ↑ membrane rigidity
Lipid–protein interface [21,52]	Maintains homeostatic αSyn–lipid interactions	↑ PS may strengthen αSyn membrane affinity	↓ PS may weaken αSyn membrane anchoring and promote cytosolic accumulation

WT: wild-type; CHOL: cholesterol; PC/PE/PS: phosphatidylcholine/ethanolamine/serine; NSF: N-ethylmaleimide-sensitive factor; CADPS2: calcium-dependent activator protein for secretion 2; AP1B1: adaptor protein complex 1 beta subunit; PICALM: phosphatidylinositol-binding clathrin assembly protein; ↑: increased; ↓: decreased. References indicate representative primary studies supporting each molecular category; mechanistic depth and translational limitations are discussed in the main text.

**Table 3:** Emerging therapeutic targets for synaptic vesicle restoration in Parkinson’s disease.

Target	Pathological Change	Therapeutic Direction	Proposed Effect
vATPase (V0a1 subunit) [16,47]	↓ Expression → ↓ Acidification	↑ V0a1 stabilization or gene therapy	↑ Neurotransmitter loading
VAMP2–SNARE complex [15,137]	↓ VAMP2 + ↑ pS129-αSyn → Fusion uncoupling	SNARE mimetics or pS129 inhibition	↑ Vesicle docking and fusion
PS/PC lipids [32,48]	↓ PS/PC → ↓ αSyn anchoring	Lipid precursors or metabolic modulators	↑ Membrane binding of αSyn
αSyn–lipid interface [63,64]	Loss of α-helix conformation → Misfolding	α-helix–stabilizing compounds	↓ Toxic oligomers, ↑ physiological function
Unsaturated lipids [20,21]	↓ Membrane fluidity → Rigid vesicles	PUFA-based dietary or pharmacological supplementation	↑ SNARE flexibility, ↑ Fusion probability
Omics–function gap [112,138]	There is no direct link between vesicle composition and output	Multimodal profiling + functional screening	Validated drug effects on SV performance

αSyn: α-synuclein; PS: phosphatidylserine; PC: phosphatidylcholine; SNARE: soluble NSF attachment protein receptor; vATPase: vacuolar-type H^+^-ATPase; PUFA: polyunsaturated fatty acids; ↑: increased; ↓: decreased; →: leads to. References provide representative support for each target, which remains conceptual and based on preclinical mechanistic evidence; feasibility and translational limitations are discussed in the main text.

## Data Availability

No new data were created or analyzed in this study. Data sharing is not applicable to this article.

## Abbreviations

The following abbreviations are used in this manuscript:

αSyn	α-synuclein
αSynBAC	Human αSyn BAC transgenic mouse
αSynKO	αSyn-knockout mouse
AP1B1	Adaptor protein complex 1 beta subunit
ATP8A1	ATPase phospholipid transporting 8A1
AZ	Active zone
BAC	Bacterial artificial chromosome
BIN1	Bridging integrator 1
CADPS2	Calcium-dependent activator protein for secretion 2
CHOL	Cholesterol
GTPase	Guanosine triphosphatase
iPSC	Induced pluripotent stem cell
lysoPC	Lysophosphatidylcholine
lysoPE	Lysophosphatidylethanolamine
lysoPS	Lysophosphatidylserine
Munc18-1	Mammalian uncoordinated-18 homolog 1
NSF	N-ethylmaleimide-sensitive factor
PICALM	Phosphatidylinositol-binding clathrin assembly protein
PC	Phosphatidylcholine
PD	Parkinson’s disease
PE	Phosphatidylethanolamine
PI(4,5)P_2_	Phosphatidylinositol 4,5-bisphosphate
PS	Phosphatidylserine
pS129	Phospho-Serine 129
Rab27B	Ras-related protein Rab-27B
SNARE	Soluble NSF attachment protein receptor
SV	Synaptic vesicle
SV2B	Synaptic vesicle glycoprotein 2B
SYNJ1	Synaptojanin-1
TEM	Transmission electron microscopy
VAMP2	Vesicle-associated membrane protein 2
vATPase	Vacuolar-type H^+^-ATPase
V_0_a1	V_0_ sector subunit a1 of vATPase
V_0_d1	V_0_ sector subunit d1 of vATPase
V_1_F1	V_1_ sector subunit F1 of vATPase
WT	Wild-type
